# Comparative Analysis of Cryotherapy Modalities Using Muscle Tissue Temperature Measurement: Cold Pack, Cold Compression, and Hyperbaric Gaseous Cryotherapy

**DOI:** 10.3390/vetsci11120613

**Published:** 2024-12-01

**Authors:** Jinyeob Baek, Jaeeon Cheon, Hyeonseo Lim, Yong Yu, Suyoung Heo

**Affiliations:** Department of Surgery, College of Veterinary Medicine, Jeonbuk National University, Iksan-si 56896, Republic of Korea; starlight223@jbnu.ac.kr (J.B.); flcl5050@jbnu.ac.kr (J.C.); lwh6079@jbnu.ac.kr (H.L.); skyyong07@jbnu.ac.kr (Y.Y.)

**Keywords:** cryotherapy, cold pack, cold compression, hyperbaric gaseous cryotherapy, dog

## Abstract

Cryotherapy is widely used in veterinary medicine to manage pain and swelling, yet optimal methods for specific tissue depths remain unclear. Cryotherapy modalities, including cold packs (CPs), cold compression (CC), and hyperbaric gaseous cryotherapy (HGC), were applied to nine beagle dogs under general anaesthesia. A custom 3D-printed guide facilitated consistent and accurate measurements of tissue temperatures at depths of 1 and 3 cm. After a 20 min application, all modalities significantly reduced the muscular tissue temperatures at both measured depths. HGC exhibited the most effective rapid temperature reduction, whereas CC displayed the most extended sustained cooling effect. In comparison, CPs showed a lower temperature reduction. The effectiveness of cryotherapy varies with tissue depth, and selecting the appropriate method can improve therapeutic outcome.

## Simple Summary

This study investigates the effectiveness of different cryotherapy methods—cold packs, cold compression, and hyperbaric gaseous cryotherapy—in reducing muscle tissue temperature in dogs. Cryotherapy is commonly used to manage pain and swelling in veterinary medicine, but the best method for different tissue depths has not been clearly established. We measured temperature changes at different tissue depths using 3D-printed guides to assess which method was most effective. We found that all three methods significantly reduced muscle temperature, but the extent and speed of cooling varied depending on the depth of the tissue and the modality used. Cold compression and hyperbaric gaseous cryotherapy were more effective at deeper tissue levels compared to cold packs.

## 1. Introduction

Cryotherapy is commonly used in veterinary medicine to manage acute injuries and postoperative pain. It can also be used in rehabilitation to mitigate muscle spasms, reduce muscle rigidity, enable more potent muscle contractions during purposeful movements, and alleviate soreness after exercise [1]. The main goal of cryotherapy is to reduce the temperature of the injured tissue and subsequently diminish its metabolic activity [2]. Cryotherapy reduces inflammation, oedema, and pain perception by inducing localised vasoconstriction and slowing the cellular metabolism [3,4,5]. Most cryotherapy procedures in veterinary medicine are adopted from human studies [6]. There is little information available on the optimal modality, application time, or duration for specific injuries or body regions in dogs.

The application of a cold pack (CP) to the skin is a classic cryotherapy method that has long been used. Commercially available CPs commonly consist of a superabsorbent polymer, ice, or a mixture of water and alcohol. There are disagreements regarding the optimal method and duration for applying a CP and its actual efficacy in human patients [7]. Despite the limited scientific evidence supporting the use of CPs, it remains a popular modality for cryotherapy because of its affordability and simplicity of use.

Cold compression (CC), a modality combining cryotherapy with intermittent pneumatic compression, has been used in patients undergoing anterior cruciate ligament reconstructive surgery [8,9]. Simultaneous compression optimises the contact between the cold source and dogs, potentially enhancing the treatment benefits. This can contribute to reductions in swelling and pain while also improving the range of motion of the affected knee [4,9]. While CC units have been utilised for human patients for a long time, commercial devices tailored specifically for canine patients are now also accessible. These canine-specific units are capable of modifying various treatment parameters, such as pressure, temperature, and time, and are equipped with custom sleeves or wraps tailored to fit diverse regions of the canine anatomy. In canine patients undergoing a tibial plateau-levelling osteotomy (TPLO), CC treatment reduces pain, swelling, and lameness [10].

Recently, various cryotherapy modalities with unique cooling mechanisms have been introduced into veterinary clinical practice. One such method is hyperbaric gaseous cryotherapy (HGC), which involves spraying CO_2_ microcrystals under high pressure and low temperatures to reduce the skin temperature through sublimation [11]. This method is presumed to lower skin temperature more significantly than traditional cryotherapy with ice, leading to a more noticeable ‘thermal shock’. Previous studies have shown that gaseous cryotherapy induces systemic skin vasoconstriction [11,12]. Previous studies in human medicine have demonstrated that HGC significantly improved pain management among orthopaedic patients [12,13,14]. Recently in veterinary medicine, it has been reported that dogs with unilateral stifle joint disease showed improved pain management and functional recovery when HGC was applied for 2 days postoperatively after stifle joint surgery [15].

Despite the prevailing consensus on the physiological effects of various cryotherapy modalities, clear guidelines regarding their application have yet to be established, and there is still a lack of definitive evidence validating the superiority of one modality over the others. To the best of our knowledge, no previous studies have compared the differences in temperature changes according to tissue depth after applying different cryotherapy modalities in dogs. The aim of this study was to compare the cooling effects of a CP, CC, and HGC modalities at different muscle depths.

## 2. Materials and Methods

### 2.1. Case Selection

This study was conducted in accordance with the guidelines and regulations of the Jeonbuk National University Institutional Animal Care and Use Committee (JBNU NON2022-049). Nine clinically healthy beagle dogs were included in this study.

### 2.2. Anaesthesia and Preparation

Each dog was pre-medicated with an intravenous (IV) injection of medetomidine (1–2 μg/kg, Domitor; Zoetis, Korea) to induce sedation. Anaesthesia was subsequently induced with alfaxalone (2 mg/kg IV; Alfaxan, Jurox, Rutherford, Australia), and the dogs were then intubated. Anaesthesia was maintained by administering sevoflurane (Sevofran, Hana Pharm Co., Seoul, Republic of Korea). The dogs were placed in dorsal recumbency on a forced-air warming system (Bair Hugger patient warming Model 750; 3 M) and covered with blankets (patient warming Model 522; 3 M) to maintain their body temperature during the study. Oesophageal temperature probes were used to monitor the core body temperature of dogs. A 20 min equilibration period was allowed before cryotherapy was applied to stabilise the core body temperature. Hair on the hindlimbs of the dogs was clipped before cryotherapy application. The skin of each limb was prepared using chlorhexidine gluconate solution and ethanol.

### 2.3. Three-Dimensional-Printed Guides Manufacturing

**Figure 1:**
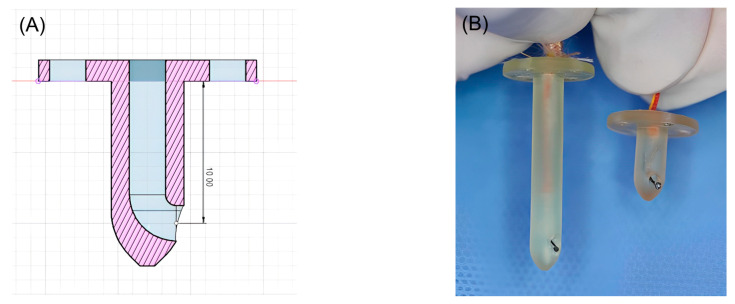
(**A**) Blueprint for 3D-printed guides capable of measuring 1cm depth. (**B**) Three-dimensional-printed guides for inserting the Type K thermocouple probe temperature sensor.

Three-dimensional-printed guides were designed using Fusion 360 software (Autodesk, San Francisco, CA, USA) to facilitate precise temperature measurements at depths of 1 and 3 cm within muscle tissue. The guide was designed to be the shape of a tack, with a blunt end. In addition, the guides were hollowed to accommodate a coiled, Type K thermocouple probe temperature sensor (Figure 1). All guides were printed using a medical-grade resin (ZMD-1000B CLEAR-SG, Zenith, Daejeon, Republic of Korea) on a resin 3D-printer (Pixel One, Zerone, Gyeonggi, Republic of Korea).

### 2.4. Calibration Procedure and Measurement Accuracy of the K-Type Thermocouple

In this experiment, a K-type thermocouple was used to measure temperature. To ensure accuracy, the thermocouple was calibrated prior to data collection. The calibration involved two reference points: a 0 °C ice–water bath and a 100 °C boiling water bath. The thermocouple probe was immersed in the ice–water mixture and then in the boiling water, each time recording the output voltage and comparing it with the expected values for these temperatures. This two-point calibration allowed us to adjust for any deviations and ensured accurate readings across the measured temperature range.

The K-type thermocouple, after calibration, had an expected accuracy of ±1 °C to ±2 °C under standard conditions, but minor deviations could still occur depending on the temperature range and environmental influences, such as thermal cycling or mechanical strain. The error range in this setup was assessed to be within ±1.5 °C based on repeated measurements at the calibration points. This precision level was considered acceptable for the purposes of this experiment, given the required temperature range and tolerances.

### 2.5. Measuring Temperature Process

**Figure 2:**
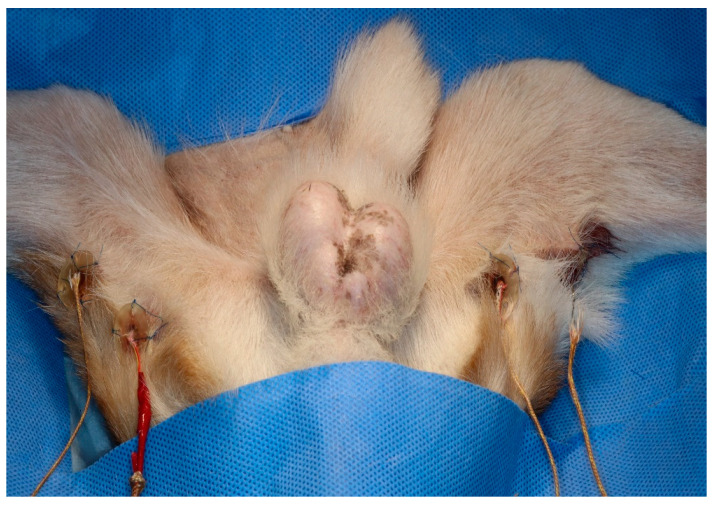
Three-dimensional-printed guides and Type K thermocouple probe temperature sensors placed in each hindlimb.

Two 3D-printed guides of different depths were secured to the skin using sutures on each hindlimb. A 1 cm skin incision was made at two points, dividing the posterior thigh into three sections. The caudal border of the gracilis muscle was then bluntly dissected. A 3 cm 3D guide and probe were inserted at the proximal site, and a 1 cm 3D guide and probe were inserted at the distal site. The sensors were inserted into the medio-caudal thigh using 3D-printed guides (Figure 2). The sensors were then connected to a digital thermometer (SDT25, SUMMIT) to determine the temperature of the muscle tissue. Oesophageal temperature probes were used to monitor core body temperature. All temperatures were recorded every minute.

### 2.6. Application of Modalities

**Figure 3:**
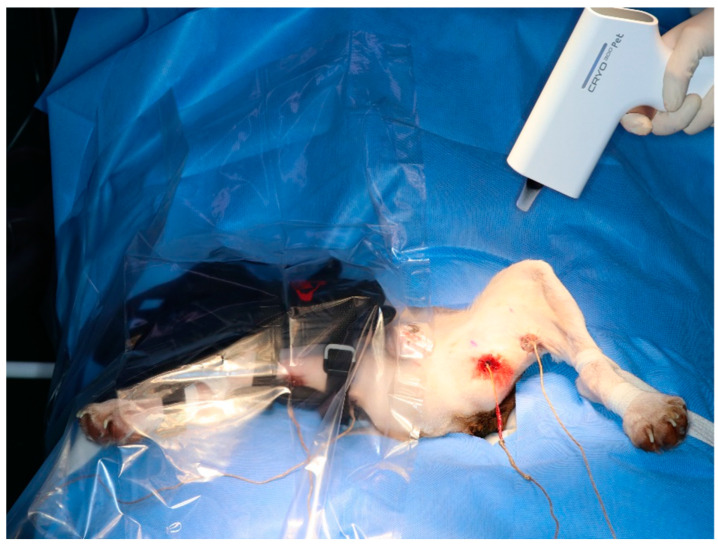
Applying a plastic film to minimise the impact of the modality on the opposite hindlimb. Cold compression on the right hindlimb and hyperbaric gas cryotherapy on the left hindlimb.

Three cryotherapy modalities were used in this study: cold packs, cold compression, and hyperbaric gas. Different cryotherapy modalities were randomly assigned to the left and right hindlimbs. Therefore, each modality was tested on the six hindlimbs of nine dogs. To minimise the interaction due to the different modalities applied to the two hindlimbs, they were separated using plastic film (Figure 3). A baseline temperature at 0 min was obtained and modalities were applied in one leg. The Contralateral modality was performed after a reduction in the baseline temperature.

The CP (MedALL hot and cold soft pack; Shanghai Industrial Co., Shanghai, China) used in this study was made of a superabsorbent polymer. The temperature of the cold pack was approximately 1.0 °C at the time of application. A CP was applied to the medio-caudal aspect of the limb, mid-way between the ischiatic tuberosity and the trochlear ridge of the femur. The CP was secured using a strap. CC was administered using a cold compression system (Companion Animal Health, USA), which comprised a control unit with an integrated pump, an adjustable wrap, and a gel pack inlaid with a hook-and-loop fastener. The gel pack was cooled in a freezer until a temperature of 1.0 °C was attained. An intermittent pressure of 50 mmHg was used. An adjustable compression wrap was applied to the medio-caudal thigh. HGC was administered using a Cryo300pet device (ISMED, Republic of Korea) which comprised the main body, an immersed tube, and a handpiece. Gaseous CO_2_ microcrystals were sprayed through a nozzle attached to the handpiece. The nozzle tip was positioned 20 cm from the skin and the medio-caudal thigh was sprayed thoroughly. The CP and CC were applied for 20 min. HGC was applied for 0 min and 10 min for a total application time of 150 s. After the application of each cryotherapy, passive rewarming with a cotton blanket was performed and measurements were collected every minute for a total of 40 min.

### 2.7. Statistics

Descriptive analyses were performed using IBM SPSS Statistics for Windows (version 29.0; IBM Corp., Armonk, NY, USA) and Excel for Microsoft 365 (Microsoft Corp., Redmond, WA, USA). A two-way repeated-measures analysis of variance (ANOVA) was used to determine whether there were any statistically significant differences in muscle tissue temperatures among the cryotherapy modalities. The analysis was conducted by dividing the data into two categories: modality application for 20 min and passive warming for 20 min, with the data being analysed at 2 min intervals. Normality was established using a Shapiro–Wilk test. A post hoc power analysis was performed using a Tukey HSD test. A *p* value of less than 0.05 was used to determine statistical significance.

## 3. Results

The mean weight of the nine dogs was 8.1 kg (range: 6.0–10.2 kg), with an average age of 1.6 years (range: 1–2 years). The body condition scores of all the dogs ranged from 4 to 5 on a nine-point scale. Three modalities were used to test the dogs, with nine limbs being tested in each modality, resulting in the 18 limbs included in the study. The set ambient room temperature was 21 °C for the duration of the study. The forced-air warming system was set at 43 °C to maintain the dog’s body temperature.

**Figure 4:**
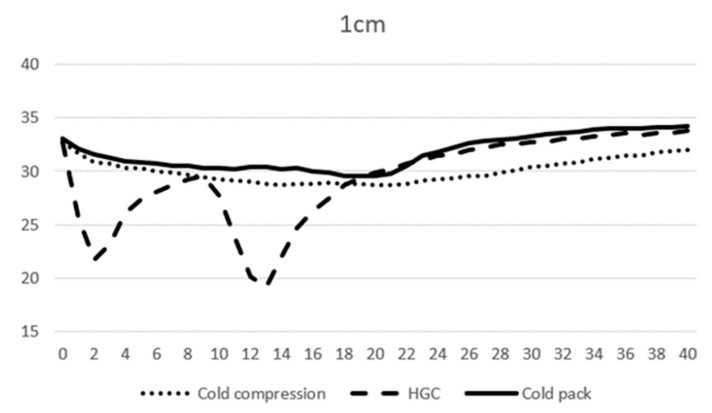
Mean temperature changes over time at a depth of 1 cm during modality application. The X-axis represents time (minutes) and the Y-axis represents temperature (°C).

**Figure 5:**
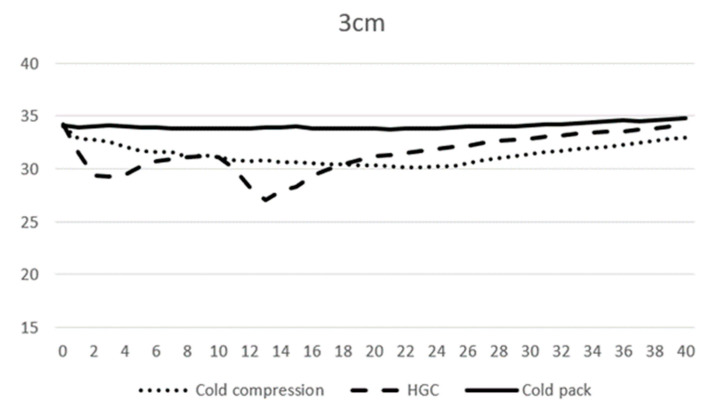
Mean temperature changes over time at a depth of 3 cm during modality application. The X-axis represents time (minutes) and the Y-axis represents temperature (°C).

At 0 min, the average temperature of muscle tissue at 1 cm depth ranged from 32.7 to 33.1 °C across all modalities, and at 3 cm depth ranged from 34.0 to 34.2 °C. At 10 min, the average temperature of muscle tissue at 1 cm depth was 29.3, 27.7, and 30.3 °C, and 31.2, 31.2, and 33.9 °C at 3 cm depth for CC, CP, and HGC, respectively (Figure 3 and Figure 4). During the first 20 min of modality application, all groups (CC, CP, and HGC) exhibited a statistically significant reduction in temperature over time at both 1 and 3 cm (*p* < 0.001). According to the post hoc Tukey HSD test, significant differences were observed at a muscle depth of 1 cm between CC and HGC (*p* = 0.037) and between the CP and HGC (*p* = 0.008). Additionally, at a depth of 3 cm, significant differences were found between the CP and CC (*p* = 0.043) and between the CP and HGC (*p* = 0.004). No statistically significant differences were found either between CC and the CP at a depth of 1 cm (*p* > 0.05) or between CC and HGC (*p* > 0.05) at a depth of 3 cm. (Figure 4 and Figure 5)

After 20 min, each cryotherapy device was removed and passive warming was performed. The mean temperature of CC, the CP, and HGC at 20 min was 28.7, 29.9, and 29.5 °C at 1 cm, respectively, and 30.3, 31.2, and 33.8 °C at 3 cm depth, respectively. After rewarming, all the groups (CC, CP, and HGC) showed an overall increase in temperature over time (Figure 3 and Figure 4). There was a statistically significant interaction between temperature and time (*p* < 0.001). The post hoc Tukey HSD test showed that the difference between CP and CC was significant at 3 cm (*p* = 0.005). Additionally, no statistically significant differences were found between any of the groups at a depth of 1 cm or between CC and HGC and CP and HGC at a depth of 3 cm (*p* > 0.05).

The cooling effect (CE) of modalities was defined as the mean change in temperature during the application of modalities.

*Cooling effect* (*CE*) = (*Temperature*
*at*
*0*
*min*) − (*Temperature*
*at*
*20*
*min*)

No significant difference in CEs at a depth of 1 cm was observed. At a depth of 3 cm, the CE was significantly higher with HGC and CC compared with the CP (*p* = 0.02). There was no significant difference between HGC and CC (*p* = 0.377) (Table 1).

The mean minimum temperature at a depth of 1 cm was 28.5, 18.2, and 29.4 °C for CC, HGC, and the CP, respectively. There was a mean temperature decrease of 4.2, 14.6, and 3.5 °C for CC, HGC, and CP, respectively. The mean minimum temperature at a depth of 3 cm was 30.0, 26.9, and 33.5 for CC, HGC, and the CP, respectively. There was a mean temperature decrease of 4.0, 7.3, and 0.7 °C for CC, HGC and the CP, respectively (Table 2).

Peak temperature reduction at a depth of 1 cm occurred at 21, 13, and 20 min in CC, HGC, and CP, respectively. Peak temperature reduction at a depth of 3 cm occurred at 22, 13, and 21 min in CC, HGC, and CP, respectively. They then ascended back toward the baseline.

At 40 min, the last temperature was measured, and the average temperature for the CC, HGC, and CP groups was 32.0, 33.8, and 34.2 °C at 1 cm, respectively, and 33.0, 34.0, and 34.8 °C at 3 cm. At 1 cm depth, CC decreased by 0.7° while HGC and CP increased by 1.0 and 1.2 °C, respectively, compared to 0 min. At 3 cm depth, CC and HGC decreased by 1.1 and 0.2 °C, respectively, while CP increased by 0.6 °C (Table 3).

There was no significant difference in the oesophageal temperature between the beagle groups over 40 min (*p* > 0.05). At 0 min, the average temperature of beagles undergoing CC-HGC was 36.2 °C, while CC-CP was 36.7 °C and CP-HGC was 36.4 °C. At the end of the temperature measurement at 40 min, CC-HGC, CC-CP, and CP-HGC were 36.1, 36.8, and 36.1 °C, respectively. When comparing the temperatures at 0 and 40 min, only the beagles on CC-CP showed an increase in temperature, whereas those on CC-HGC and CP-HGC showed a decrease in temperature.

## 4. Discussion

To our knowledge, the present study is the first to report temperature changes according to tissue depth resulting from the application of cryotherapy modalities in dogs. This study also introduces a novel method for measuring temperature based on tissue depth: a 3D-printed guide designed to measure temperatures at specific depths.

Results of this study indicated that the application of all modalities for 20 min significantly reduced the temperature of muscular tissue at 1 and 3 cm in beagles following general anaesthesia. Previous studies related to CP have determined that the superficial tissue undergoes the largest decrease in temperature [6,16,17,18]. In this study, the reduction in tissue temperature was also strongly influenced by the depth of the treated tissue. The temperature changes at deeper depths for the CP, CC, and HGC were similar to the trends of temperature differences in deeper tissues reported in other studies [6,16,17,18]. In this study, the CP showed an average temperature reduction of 3.5 and 0.7 °C at depths of 1 and 3 cm. In Ralph et al.’s study, commercial-grade frozen gel packs were applied for 20 min and showed an average temperature reduction of 6.45 °C at a depth of 1 cm [6]. Janas et al. used cold packs consisting of two parts chipped ice to one part isopropyl alcohol and showed an average temperature reduction of 2.3 °C at a depth of 1 cm and 1.7 °C at a depth of 3 cm after 20 min of application [17]. These temperature differences between the studies are expected to be due to the temperature at which the pack was cooled prior to application, the composition of the pack utilised, and the method of contact with the pack. This suggests that clinical outcomes may vary based on the differences in these factors.

In this study, all modalities exhibited rapid cooling at a depth of 1 cm followed by gradual cooling at a depth of 3 cm. The second law of thermodynamics explains the rapid and profound cooling of the superficial tissue [6,17]. The second law of thermodynamics dictates that heat is inevitably transferred from a region with a higher temperature to one with a lower temperature. The cooling effect of cryotherapy on tissues does not directly transfer cold into the tissue; instead, heat from the tissue is transferred into the cooling modalities. Deeper tissues are not directly affected by the cryotherapy modality; therefore, they cool by transferring heat to the superficial layers of the tissue. The thickness of the tissue affects the time required for heat transfer, as do the tissue thickness, contact area, weight of compression, starting temperature, and thermal conductivity. Therefore, for cryotherapy to be clinically meaningful, it may need to be applied for longer durations when targeting deeper tissues to achieve a significant temperature drop.

The CE was measured by comparing the temperatures at 0 min and 20 min. At a depth of 1 cm, a significant decrease in temperature was observed in all three modalities. At a depth of 3 cm, CC and HGC demonstrated remarkable CEs, showing significant temperature changes, whereas CP showed only subtle changes.

HGC demonstrated the lowest mean minimum temperature at both 1 and 3 cm depths compared to CC and the CP. Additionally, HGC achieved the mean minimum temperature faster than the other modalities. At 1 cm depth, the temperature decrease with HGC was 14.6 °C, which is 3.4 times greater than CC and 3.9 times greater than the CP. At 3 cm depth, the temperature decrease with HGC was 7.3 °C, which is 1.9 times greater than CC and 18.9 times greater than CP. HGC operates by utilising liquid carbon dioxide that is deposited on the skin and transformed into a solid phase (dry ice). Subsequently, the dry ice sublimates into carbon dioxide gas due to atmospheric pressure. This sublimation process results in a greater amount of heat absorption compared with the convective cooling provided by a CP or CC. As a result, HGC exhibited a more rapid and substantial decrease in skin temperature.

The temperature window for significantly reducing tissue metabolism is reported to be between 5 °C and 15 °C [19]. However, achieving such temperatures in living animals poses a substantial challenge, as these findings are primarily based on studies conducted with excised tissues. Nevertheless, the primary effect of cryotherapy extends beyond merely reducing tissue metabolism. Even achieving mild to moderate decreases in muscle temperature can yield considerable clinical benefits, including pain relief, inflammation suppression, and protection against further tissue damage [20].

Previous studies have suggested that greater and faster cooling of tissue temperature leads to better clinical outcomes. A cooler cryotherapy technique yields greater physiological and biological effects, such as decreases in edoema, pain, muscle spasms, and inflammation. Rapid cooling of cryotherapy is advantageous because the extent of secondary injury is minimised by the rapid decrease in cellular metabolism [21,22].

The observed properties of HGC indicate that it possesses effective heat abstraction capabilities, making it a viable option for achieving localised analgesia, reducing nerve-conduction velocity, and modulating cell metabolism [11]. According to Van’t Hoff’s rule, for every 10 °C decrease in tissue temperature, the chemical reaction rate is reduced by a factor of 2 to 3 [23]. This principle forms the foundation for the application of cryotherapy immediately after acute injuries and suggests that, among the three modalities in this study, HGC is likely to be the most effective. 

The findings of this study indicate that, after a 20 min application of cryotherapy, CC exhibited the longest duration of temperature decrease compared to the other modalities at both 1 and 3 cm depths. This suggests that CC provides a sustained and prolonged cooling effect on tissues. This may be due to several factors, including the compression applied by the cooling material, which enhances heat transfer from the tissue. In contrast, HGC showed a trend of temperature increases immediately after spraying ended. This could be attributed to factors such as the rewarming effects or cooling mechanisms employed by HGC. The sustained cooling effect demonstrated by CC may have clinical significance, potentially offering a more pronounced residual cooling effect than other modalities such as HGC and CP. This sustained cooling effect can be particularly beneficial in situations where long-lasting therapeutic effects, such as localised analgesia or reduction in tissue metabolism, are desired.

When comparing the temperatures at the beginning (0 min) and end (40 min), it was observed that, at a depth of 1 cm, both CP and HGC returned to above baseline temperatures during the course of the study. These results suggest that CC had the most prominent total cooling at a 1 depth of 1 cm. At a depth of 3 cm, CP returned to baseline, whereas CC and HGC exhibited lower temperatures at 40 min than at the beginning. The findings at 1 and 3 cm depths further highlight the differential cooling responses among the modalities. This suggests that CC and HGC have the potential to provide greater total effects of cooling in deeper tissues than CP.

Previous human studies have reported HGC application times of 90 and 120 s for the knee [12,14]. In one previous study on dogs, HGC was applied for 90 s after TPLO surgery, with an additional 30 s if the skin temperature did not reach 17–18 °C [15]. In this study, HGC was applied twice at 0 and 10 min of the experiment, each time for 150 min, as recommended by the manufacturer. It is important to exercise caution when applying prolonged cryotherapy, as it may lead to tissue damage due to sustained vasoconstriction and the resulting hypoxaemia in the localised area. However, there is currently no standardised recommended duration or frequency in terms of HGC use in dogs. The optimal application parameters, including the time and number of HGC sessions, should be determined based on various factors, such as the patient’s condition, the specific area of application, and treatment goals. Further studies are needed to establish clear criteria and standardised guidelines for the use of HGC in dogs.

In this study, we designed a 3D-printed guide that facilitates the insertion of a sensor to a predetermined depth within the tissue for accurate temperature measurement at a specific tissue depth. Previous studies have utilised commercially available needle probes for depth-specific temperature readings, which were then secured in place using either a plastic sleeve or tape [6,17]. However, these methods are prone to shortcomings, notably the potential for incomplete fixation of the probe, leading to inconsistencies in measurement depth during the experiment. To mitigate this challenge, our 3D-printed guide was designed with a suture hole, permitting it to be firmly secured to the skin using nylon sutures. This approach ensured the consistent position and depth of the temperature sensor throughout the experiment, thereby enhancing the reliability of the obtained temperature measurements.

This study has a few limitations that need to be considered. First, the experiment was conducted under general anaesthesia, which could potentially influence the outcomes due to the possible effects of anaesthesia on the thermoregulatory mechanisms of an animal. Therefore, the results may not directly translate to a clinical setting in which anaesthesia is not always present.

Second, the experimental design involved the application of different cryotherapy modalities to both hindlimbs in a single subject. This approach has the potential for systemic effects, which may affect the isolated study outcomes for each modality. While we attempted to minimise this interference using a plastic film to separate the left and right hindlimbs, we cannot completely exclude the possibility that a given modality might have exerted some influence on the opposite hindlimb, thereby impacting the experimental results.

Another potential limitation is the use of a forced-air warming system to maintain the core body temperature of the patient during the experiment. The introduction of warm air from this system could potentially alter the effectiveness of the different cryotherapy modalities, thereby affecting the results of this study.

Finally, the experiments were conducted solely on beagle dogs within a specified weight range. This raises questions regarding the applicability of our findings to other breeds or dogs with significantly different weights. Our results may vary when applied to different breeds or weight categories, and further studies that include diverse species and weight ranges are required to provide a more comprehensive understanding.

## 5. Conclusions

In conclusion, we achieved accurate and reliable measurements of the tissue temperatures using a 3D-printed guide. Comparing the three cryotherapy modalities, HGC proved to be the most effective in reducing tissue temperature. Furthermore, CC demonstrated the most significant sustained cooling effect, thereby extending the therapeutic benefits of cryotherapy.

## Figures and Tables

**Table 1:** The mean cooling effect (CE) for three modalities, expressed in °C. These values represent the average of six measurements for each modality along with the standard deviation.

	CE (1 cm)	CE (3 cm)
CP	3.52 ± 1.39	0.37 ± 1.02
CC	4 ± 2.29	3.7 ± 1.93
HGC	2.93 ± 0.90	3.05 ± 1.39

**Table 2:** Maximum reduction in temperature for three modalities, expressed in °C. These values represent the average of six measurements for each modality along with the standard deviation.

	1 cm	3 cm
CP	3.5 ± 1.4	0.7 ± 0.6
CC	4.2 ± 2.1	4 ± 1.8
HGC	14.6 ± 1.0	7.3 ± 1.9

**Table 3:** Temperature gap between 40 min and 0 min (40 min minus 0 min) for three modalities, expressed in °C. These values represent the average of six measurements for each modality along with the standard deviation.

	1 cm	3 cm
CP	1.2 ± 2.4	0.6 ± 1.2
CC	−0.7 ± 1.5	−1.1 ± 1.1
HGC	1.0 ± 1.0	−0.2 ± 1.3

## Data Availability

The data presented in this study are available from the corresponding author upon reasonable request.
